# Comparing Quantitative Trait Loci and Gene Expression Data

**DOI:** 10.1155/2008/719818

**Published:** 2008-09-16

**Authors:** Bing Han, Naomi S. Altman, Jessica A. Mong, Laura Cousino Klein, Donald W. Pfaff, David J. Vandenbergh

**Affiliations:** ^1^RAND Corporation, Santa Monica, CA 90407, USA; ^2^Department of Statistics, The Pennsylvania State University, University Park, PA 16802, USA; ^3^Department of Pharmacology & Experimental Therapeutics, University of Maryland School of Medicine, Baltimore, MD 21201, USA; ^4^The Laboratory of Neurobiology and Behavior, Rockefeller University, New York, NY 10065, USA; ^5^Department of Biobehavioral Health, The Pennsylvania State University, University Park, PA 16802, USA; ^6^Center for Developmental and Health Genetics, The Pennsylvania State University, University Park, PA 16802, USA

## Abstract

We develop methods to compare the positions of quantitative trait loci (QTL) with a set of genes selected by other methods, such as microarray experiments, from a sequenced genome. We apply our methods to QTL for addictive behavior in mouse, and a set of genes upregulated in a region of the brain associated with addictive behavior, the nucleus accumbens (NA). The association between the QTL and NA genes is not significantly stronger than expected by chance. However, chromosomes 2 and 16 do show strong associations suggesting that genes on these chromosomes might be associated with addictive behavior. The statistical methodology developed for this study can be applied to similar studies to assess the mutual information in microarray and QTL analyses.

## 1. Introduction

The association between a complex phenotypic trait and genetic markers on the chromosomes can be detected through statistical analysis, leading to the identification of quantitative trait loci (QTL)—regions of the chromosomes that appear to be associated with the phenotype. Quantitative trait loci (QTL) are expected to be associated with the genes controlling some aspects of the phenotype. One mechanism by which a gene might be associated with the trait is through altered transcription which is easily measured by microarray analysis. Microarrays have the ability to measure a large percentage of the genes in the genome, and this assessment parallels the genome-wide scan performed by QTL methods.

Several investigators have considered combining QTL and microarray data for studying a genetic trait. For example, Wayne and McIntyre [1] proposed a way of identifying candidate genes based on both QTL mapping and microarray data, where loci for an interesting quantitative trait were primarily used for prescreening genes. A parallel microarray study focused on the filtered gene list and identified differentially expressed genes related to the same trait. When type I error is particularly emphasized, a QTL analysis prescreen can be used to greatly reduce the number of genes under consideration, and hence reduce the effect of multiple testing. When the objective of the research is gene discovery, a combined analysis can focus attention on genes and QTL most likely to be associated with the trait. Fischer et al. [2] developed a web-based software tool for combined visualization and exploration of gene expression data and QTL. The methodology developed in this work is complementary to the analyses that can be performed on the GeneNetwork website (WebQTL, http://www.genenetwork.org/), which allows assessment of the relationship between gene expressions and QTL in recombinant in bred mice [3].

Comparing QTL and microarray data is not completely straightforward. The estimated range of QTL positions is generally wide, containing many possibly interesting genes. In addition, QTL analysis may also miss some interesting genes [1]. The high level of experimental errors and limitations in microarray data analysis inevitably introduce mistakes in the identification of relevant genes. Finally, QTL studies include the entire genome including noncoding regions, while microarray studies seldom include the entire genome.

Further problems arise when we try to associate phenotypes with gene expressions in specific tissues. While the association is direct if the tissue from which transcription is assayed defines the phenotype, unanticipated associations can arise if the tissue indirectly regulates the phenotype—for example, bone strength may be regulated through physical activities regulated by the brain. Alternatively, association can arise through pleiotropic expression of the gene in a tissue not included in the expression study but in which the gene plays a role in the phenotype. In addition, the association between a phenotype and a tissue may depend on ephemeral conditions that may not be present when the tissue was collected for the microarray study or on a small percentage of cells in the organism, which may be masked by bulk tissue preparation.

In this paper, we suggest methods to examine the strength of association between a set of genes and a set of intervals along the genome. The methods identify genomic intervals with unusually high numbers of genes in the set, and hence genes and genomic intervals which are more likely to be associated. The methodology is not tied to the data source. Typically, the genes will be chosen from a gene expression study, while the genomic intervals will be QTL. The methods by which the regions and genes are selected affect the biological interpretation of the results, but not the statistical assessment of association.

## 2. Methods

Our objective is to determine whether genomic intervals on the chromosome (usually QTL) and gene loci (often selected from gene expression studies) are collocated on the chromosome. This requires matching of gene loci to genomic intervals and then measuring the strength of association. Quantitative trait loci (QTL) are often measured in recombination distance, while gene loci are usually reported as physical distances. In this section, we first discuss conversion between recombination and physical distances. We then discuss 2 approaches to measure strength of association.

### 2.1. Converting Recombination Distance to Physical Distance

Quantitative trait loci (QTL) are reported in centimorgans (cM), which measure recombination frequencies between markers on a chromosome. Gene locations are usually measured by the physical distance in base pairs (bp) or megabase pairs (1 Mb = 10^6^ bp). To match QTL sets and gene sets, we adopted the embedded conversion tool in expressionview [2] to estimate physical distances from cM, using a subset of genes for which both distances are available. The “smoothing window” technique used in expressionview essentially applies piecewise regression. In regions in which expressionview appeared to give poor estimates, we also used polynomial regression to estimate physical distance from cM by using genes for which both measures are available. Any QTL with a resulting span that extends beyond the end of a chromosome is truncated.

### 2.2. Measures of Association Based on Completeness and Accuracy

For convenience, we denote a set of QTLs, such as drug abuse QTLs, by *Q*, and a set of genes, such as the NA genes, by *G*. A natural approach is to consider the percentage of genes in *G* covered by at least one QTL in *Q*. The association between *Q* and *G* is strong if this number is big. This quantification reflects the “completeness” of *Q* in terms of covering *G*. A complementary approach is to consider whether each QTL in *Q* covers at least one gene in *G*. If a QTL in *Q* covers no genes in *G*, it is called “empty”; otherwise it is “nonempty.” The association between *Q* and *G* is strong when the percentage of empty QTL is small. This quantification reflects the “accuracy” of *Q* in terms of covering *G*.

If *Q* is strongly associated with *G*, we expect both completeness and accuracy to be high. However, the two methods do not necessarily give the same result because they are measuring complementary aspects of an association. As quantitative trait loci (QTL) are added to *Q*, we expect higher completeness because the QTL in *Q* cover more segments of each chromosome. However, if these quantitative trait loci (QTL) are unrelated to *G*, we expect many of them to be empty. Similarly, as genes are added to *G*, we expect higher accuracy because selected genes are found in more locations. But if the additional genes are unrelated to *Q*, we expect few of them to be covered by *Q*.

To account for the effect of increasing the size of *Q* or *G*, we need to develop a combined measure on both completeness and accuracy together to answer the question: is the overall association strong? We propose an appropriately weighted average of completeness and accuracy which penalizes for adding spurious QTL or genes to the sets.

Let *N* be the number of genes in *G*, *M* be the number of QTL in *Q*, *n* be the number genes in *G* covered by *Q*, and *m* be the nonempty QTL in *Q*, then completeness *C* = *n*/*N*, and accuracy *A* = *m*/*M*. We define the combined measure of association as

(1) $$S=\frac{C}{M}+\frac{A}{N}.$$

The weight is chosen to diminish the effect of matching by chance. When *M* increases, more of the genome will be covered by *Q*; we compensate by dividing by *M*. We weight accuracy by 1/*N* to penalize for increasing the size of *G* in the same fashion.

The limiting behaviors of the combined measure *S* satisfy the need to differentiate a strong association from a noisy one in which matching results by chance. Let *s* be the number of genes in *G* which really match some QTL in *Q*. Correspondingly, let *r* be the number of QTL in *Q* that really match some genes in *G*. Note *r* usually is not equal to *s*. Besides the true matching relationship, every gene has probability *p* = *p*(*M*) of being covered by *Q* leading to *u* genes which are covered by chance. On the other hand, every QTL has a probability 1 − *q* = 1 − *q*(*N*) of being empty with respect to *G*, so that it has a probability *q* of being “nonempty” leading to *v* QTL which are nonempty by chance. Completeness can be written as

(2) $$C=\frac{s+u}{N}.$$

Thus, the expectation of *C* is straightforward:

(3) $$\text{E}C=\frac{s+(N-s)p}{N}.$$

Similarly,

(4) $$\begin{array}{cc} A & =\frac{r+v}{M},\\ \text{E}A & =\frac{r+(M-r)q}{M}. \end{array}$$

Then,

(5) $$\text{E}S=\frac{r+s+(N-s)p+(M-r)q}{MN}.$$

Consider the following limiting circumstances: (1) (perfect match) when *s* → *N* and *r* → *M*, E*S* monotonically increases to the limit (*M* + *N*)/*MN*; (2) (totally random) when *s* → 0 and *r* → 0, E*S* monotonically decreases to the limit (*Np* + *Mq*)/*MN*; (3) (*G* has spurious genes) when *N* → *∞* and *M* is fixed, notice *q* = *q*(*N*) → 1 in this case, E*S* will converge to *p*/*M*; (4) (*Q* has spurious QTL) when *M* → *∞* and *N* is fixed, notice *p* = *p*(*M*) → 1 in this case, E*S* will converge to *q*/*N*. From the above, it can be concluded that the combined measure *S* will approach its maximum when a perfect match arises and decrease when the association weakens in some respect.

## 3. Statistical Tests for Accuracy and Completeness

Until the biology is fully understood, we cannot be certain if the association between *Q* and *G* is due to chance. We, therefore, want to study the pair of hypotheses H_0_: there is no stronger association between QTL and genes than expected by chance, that is, we cannot benefit from combining the results; H_a_: the association is stronger than expected by chance. In this section, we determine the statistical significance of the observed levels of association by comparing with the null distribution of completely random association determined by simulation. Random selection of QTL is not readily done as selection of random intervals along the chromosomes because we do not know the location of all possible QTL. Hence random selected intervals are unlikely to model the true distribution of QTL. However, since the physical locations of all genes on the microarray are known, random sets of genes are readily created by choosing genes at random and considering the null distributions of completeness or accuracy of the QTL sets with respect to these randomly chosen genes.

To assess the strength of association between a QTL set *Q* and a gene set *G* of size *N*, we compute the completeness and accuracy of *Q*. We then select genes at random from all the genes represented on the microarray. The simplest way to do this is to select *N* genes at random from the array. However, since there is considerable variability in the percentage of tissue-specific genes on each chromosome and since the QTL may not be randomly distributed among chromosomes, we can also consider selecting *N*_*i*_ genes from the *i*th chromosome, where *N*_*i*_ is the number of genes in the gene set on the chromosome. We call the latter method the conditional method because the random selection strategy is conditional on the number of genes in *G* on each chromosome. By contrast, the former method, which selects genes completely at random, is called the unconditional method. It is not entirely clear when the conditional and unconditional methods are more appropriate or powerful. When the gene set and quantitative trait loci (QTL) are distributed across all of the chromosomes, we might expect that the conditional method will be more powerful as well as more precise. However, when there are chromosomes with no genes in *G*, the unconditional method should be more powerful, as it takes into account the probability that QTL and genes in the gene set may not be on the same chromosome, whereas the conditional method uses only information about the chromosomes which include genes in *G*. Conversely, when there are chromosomes with no QTL in *G*, the conditional method may be more powerful because genes selected from these chromosomes will not contribute to completeness or accuracy under conditional random sampling strategy.

By repeatedly selecting gene sets at random either conditionally or unconditionally and computing the completeness and accuracy for *Q*, an unconditional or conditional null distribution is then simulated. The *P*-values for the observed completeness *C*, accuracy *A*, and combined measure *S* are the percentages of simulated datasets for which the simulated *C*, *A*, and *S* are as strong as or stronger than the corresponding observed values. The estimated *P*-values are displayed in Table 1 based on 10000 sets of randomly selected genes. Since the *P*-values are based on count data, we also consider 3 continuity corrections which differ in how the rejection region includes the observed counts. The simulations took about 296 seconds on a 2.8 GHz computer with 2 GB of RAM running Windows XP.

As part of the simulation, we can also compute the distribution of number of genes covered by each QTL. Percentiles of this distribution can be used to identify quantitative trait loci (QTL) that have unusually large coverage on the observed data and are thus more likely to be associated with genes implicated in the QTL condition.

### 3.1. Chi-Square Tests of Association

The count of nonempty QTL *m*_*i*_ and covered genes *n*_*i*_ of each chromosome can be used to construct a chi-square-type of test for either accuracy or completeness. Using chromosomes as the natural category, we define the chi-square (*𝒳* ^2^) test statistic as

(6) $$\begin{array}{cc} {𝒳}^{2} & =\overset{T}{\underset{i=1}{\sum }}\frac{(X_{i}-\text{E}X_{i}{)}^{2}}{\text{E}X_{i}}\;\;∼\;{𝒳}_{T-1}^{2}\;\text{ under }{\text{H}}_{0}\text{:}\\ & \text{the link is no different from random,}\\ X_{i} & =n_{i},m_{i}, \end{array}$$

where E*X*_*i*_ under H_0_ can be estimated by random sampling genes, and *T* is the total number of chromosomes. Under H_0_: the link is no different from random; E*X*_*i*_ is the same as expected counts when genes are selected at random. For a given set of QTL, we can repeatedly sample random genes. The average of observed counts of nonempty QTL or covered genes is a consistent estimator for E*X*_*i*_ and is quite accurate since we repeat sampling many times. A true association between QTL and genes will increase the observed counts which result in a larger chi-square test statistic. As well, the chi-square test provides us with additional insight in identifying potential candidate differentially expressed genes, if quantitative trait loci (QTL) are mapping the same or very similar quantitative traits as the partner microarray study. On the one hand, a chromosome that has a large positive value suggests a region of strong association with the gene set, and hence lends support to the hypotheses that the QTL and the covered genes are associated with the trait of interest. Conversely, a chromosome for which *X*_*i*_ − E*X*_*i*_ is a small positive value or a larger negative value suggests that the QTL and the covered genes may not be associated with the trait. Thus, association between the *Q* and *G* can also be used to select QTL and genes which are more likely to be of interest.

### 3.2. Materials

We apply our methods to a set of mouse QTL identified from the literature and a set of mouse genes identified from a microarray study. First, we identified a set of 120 QTL associated with drug abuse behaviors in mice [4] from the Mouse Genome Informatics (MGIs) database (http://www.informatics.jax.org). A set of 166 genes that are preferentially expressed in the nucleus accumbens (NA) region of the mouse brain (the NA genes) was determined from a microarray study of brain tissues [5] using Affymetrix MG-U74Av2 arrays. This array contains about 1/3 of the coding genes in the mouse genome. Briefly, the NA genes were identified as being expressed at least 1.5 fold higher in the nucleus accumbens compared to two other brain regions, the medial basal hypothalamus and preoptic area, in one-day-old C57BL/6J mouse pups. The study did not include replication, so the statistical significance of the expression differences cannot readily be assessed; association of these genes with QTL, therefore, becomes an important tool to help assess the biological significance of the observed differential expression.

The two brain regions used for comparison are physically close to the NA on the ventral surface of the brain but are largely derived from a different embryonic region of the brain (diencephalon compared to telencephalon for the NA). The NA plays an important role in drug abuse-related behavior. Our primary objective was to determine if the QTL and gene expression studies are detecting a set of genes in common that might be related to drug abuse behaviors. However, because the NA genes are selected based on their expression in a selected region of the brain, rather than their direct involvement in drug abuse behaviors, we need to be cautious about the biological interpretation of an association between the QTL and the gene set.

### 3.3. Results

**Figure 1:**
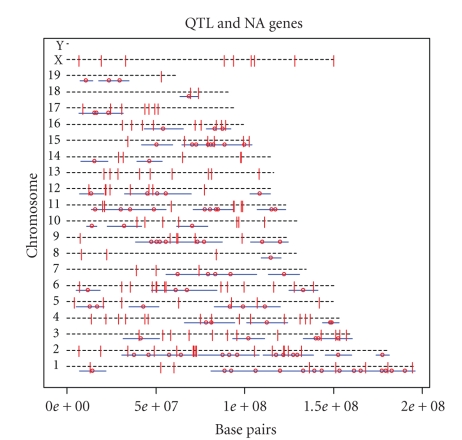
Combined visualization of QTL and NA genes. The short discrete horizontal segments are the spans of the QTL defined as +/−5 centimorgans (cM) from the peak QTL position. The small circles in the center of every segment are the peak positions of the QTL. Finally, the vertical lines are the NA genes.

Figure 1 shows the correspondence between the set of QTL and the set of NA genes. The long horizontal dashed lines are numbered to represent the mouse chromosomes. No data were available regarding gene expression or QTL on the very short Y chromosome. The positions of the NA genes were determined using the Affymetrix metadata for the MG-U74Av2 (version 1.10.0) array provided in the Bioconductor suite in R [6].

No obvious matches between the QTL set and the NA genes can be seen in Figure 1. The visual impression does not support a strong association between QTL and genes. The observed completeness is 44.6%, and the observed accuracy is 64.2%. The observed *S* is .0076, compared with the theoretical maximum for *S* of 0.014. *p*(*M*), and *q*(*N*) can be estimated from the simulation and hence we can estimate the three local minima under limiting circumstances 2, 3, and 4 discussed in Section 2.

Table 1 displays the comparison of *S* values under both randomization and limiting circumstances. Although it is far from the maximum, the observed *S* is above all the estimated local minima for genes selected at random.

The *P*-values for completeness, accuracy, and *S* are in Table 2. The *P*-values for the conditional tests are smaller than those for the unconditional tests, which is expected since there are chromosomes with NA genes but no QTL. However, there is no indication that there is significant association between the NA genes and the QTL set.

The results of the chi-square test are in Table 3. The unconditional chi-square test supports the hypothesis that the completeness is higher than expected by chance, but accuracy is only marginally higher than expected by chance—that is, more genes are covered than expected, but the number of QTL containing genes in *G* is about what is expected by chance. For example, chromosomes 2 and 16 have much higher positive values than expected under unconditional sampling which suggests that the genes on these two chromosomes are more likely to be associated with the drug abuse trait, and the QTL on these two chromosomes are more likely to be associated with the NA region than genome-wide average.

Two genes on chromosome 2, Pax6 (paired box gene 6) and Pcna (proliferating cell nuclear antigen) and one on chromosome 16, Tiam1 (T-cell lymphoma invasion and metastasis 1) lie under QTL and have some relationship to addictive behavior traits. Given that differences in gene expression were used as the criterion for selecting genes, it is not unexpected that two of these genes, Pax6 and Tiam1, are transcription factors. Pax6 plays a role in differentiation of precursor cells into neurons and glia and is altered in models of fetal alcohol syndrome [7]. Tiam1 is known to regulate growth cone morphology, a process that can be altered by drugs of abuse [8].

We note that chromosome 18 includes a gene that is just outside a QTL region. To better understand the effect of a small change in the QTL definition, we increased the length of the QTL by 0.5 Mb, which is less than a 5% increase in length for most QTL. Although most of the measures of association and their statistical significance are scarcely affected, the *P*-value for the conditional chi-square test of completeness changed from 0.910 to 0.182. This finding further supports the conjecture that the completeness in our study may be higher than expected by chance. On the other hand, this also demonstrates that the proposed chi-square test is not robust against the disturbance in data than the previous simulation-based tests.

A similar chi-square test can be used to determine if a particular QTL covers more genes than expected by chance. For example, the 3 QTL on chromosome 16 and the QTL on chromosome 18 all cover more genes than expected by chance, but none of the QTL on chromosome 19 are more accurate than expected by chance.

## 4. Conclusion and Discussion

A strong association was expected between the NA genes and the drug abuse QTL, but this hypothesis was not fully supported by the data. A possible reason is that there are a number of QTL not associated with NA genes. In addition, this large set of QTL is associated with diverse drug abuse traits, including both physiological and psychological factors, and hence may be associated with multiple brain regions. As well, because the genes were selected from an unreplicated study based on fold change, we can expect large false detection and nondetection rates. A large false detection rate implies that the NA genes likely include several genes which are not associated with the NA and are, therefore, similar to genes selected at random. We can also expect a large number of false nondetections which should induce a correspondingly large impact on the power to detect the accuracy of the QTL.

Completeness, accuracy, and the combined measure have been proposed as methods to determine whether a set of QTL and a set of genes are associated. The statistical significance of the association can be estimated by selecting sets of genes at random from the population of genes from which the gene set was determined. When *P*-values or other measures of strength of association between the trait of interest and the QTL are available or when some QTL are of particular interest a priori, we might consider weighting the measures of accuracy so that a penalty is incurred if a QTL highly associated with the trait is empty, and a gain is incurred if a gene is covered by a highly associated QTL. Weights on the QTL can readily be incorporated in the simulations required to estimate the *P*-values because the QTL are fixed in the simulation. Weights on the genes are less readily handled because weights are not available for genes selected at random. In this study, we did not use weights—however, several QTL are represented more than once in the QTL set, because the same QTL was identified from multiple sources. For example, on chromosome 1, there are 3 QTL identified in 3 studies investigating different phenotypes and located identically. These QTL may be caused by the same gene or by nearby genes. The chromosome locations covered by these QTL have higher weight in the computation of both accuracy and completeness. Removing duplicate QTL did not alter the conclusions for this set of genes and QTL.

The chi-square test provides a simple method to identify QTL and genes from the gene set that are most highly associated. A QTL that covers more genes than expected by chance is likely to include a cluster of genes from the gene set, which lends credence to the hypotheses that the QTL and the covered genes are associated with the trait of interest. Some examples of this have been detected in these data, for example, the QTL on chromosomes 16 and 18 are more accurate than expected under unconditional sampling. The chi-square tests appear to be more sensitive than the other suggested tests to small changes in the definition of the QTL, particularly when a chromosome has only a small number of QTL, and the change in definition changes *m*_*i*_ or *n*_*i*_. The other tests of accuracy and completeness or the combined measure appeared to be more robust against changes of the same magnitude.

The data and R code can be accessed from http://www.stat.psu.edu/~naomi/QTLsoftware/.

## Figures and Tables

**Table 1:** Estimated limiting extrema of combined measure *S*.

Limiting case defined in Section 2	Conditional	Unconditional
Random selection	0.00406	0.00389
Spurious genes	0.0069	0.00160
Spurious QTL	0.00237	0.00229
Theoretical maximum *S*	.0144	
Observed *S*	.00758	

**Table 2:** Simulated one-sided *P*-value for the hypothesis H_0_: the association is not stronger than expected by chance.

Measure	Observed	Definition of *P*-value	Conditional	Unconditional
*C* (Completeness)	0.446	*p* (>observed)	0.144	0.329
		1/2*p* (# =observed) + *p* (>observed)	0.168	0.357
		*p* (≥observed)	0.192	0.385

*A* (Accuracy)	0.642	*p* (>observed)	0.328	0.544
		1/2*p* (# =observed) + *p* (>observed)	0.361	0.576
		*p* (≥observed)	0.395	0.608

*S* (Combined)	.0076	*p* (>observed)	0.213	0.441
		1/2*p* (# =observed) + *p* (>observed)	0.229	0.459
		*p* (≥observed)	0.245	0.478

**Table 3:** *P*-value from the chi-square test for the hypothesis H_0_: the association is not different from expected by chance.

Chi-square test	Conditional	Unconditional
*P-value* (Completeness)	0.902	0.041*
*P-value* (Accuracy)	0.608	0.246

*Significant at 5% level.
